# An Assay System for Point-of-Care Diagnosis of Tuberculosis using Commercially Manufactured PCB Technology

**DOI:** 10.1038/s41598-017-00783-8

**Published:** 2017-04-06

**Authors:** Daniel Evans, Konstantinos I. Papadimitriou, Louise Greathead, Nikolaos Vasilakis, Panagiotis Pantelidis, Peter Kelleher, Hywel Morgan, Themistoklis Prodromakis

**Affiliations:** 1 Nanoelectronics & Nanotechnology Research Group, School of Electronics and Computer Science, University of Southampton, Southampton, SO17 1BJ UK; 2 Centre for Immunology and Vaccinology, Division of Infectious Diseases, Department of Medicine, Imperial College London, London, UK; 3 Infection and Immunity, Imperial College NHS Trust, Charing Cross Hospital, London, UK

## Abstract

Rapid advances in clinical technologies, detection sensitivity and analytical throughput have delivered a significant expansion in our knowledge of prognostic and diagnostic biomarkers in many common infectious diseases, such as Tuberculosis (TB). During the last decade, a significant number of approaches to TB diagnosis have been attempted at Point-of-Care (PoC), exploiting a large variation of techniques and materials. In this work, we describe an electronics-based Enzyme-Linked ImmunoSorbent Assay (eELISA), using a Lab-on-a-Printed Circuit Board (LoPCB) approach, for TB diagnosis based on cytokine detection. The test relies upon an electrochemical (amperometric) assay, comprising a high-precision bioinstrumentation board and amperometric sensors, produced exclusively using standard PCB manufacturing processes. Electrochemical detection uses standard Au and Ag electrodes together with a bespoke, low-power, multichannel, portable data-acquisition system. We demonstrate high-performance assay chemistry performed at microfluidic volumes on Au pads directly at the PCB surface with improved limit of detection (~10 pg/mL) over standard colorimetric ELISA methods. The assay has also been implemented in plasma, showing the utility of the system for medical applications. This work is a significant step towards the development of a low-cost, portable, high-precision diagnostic and monitoring technology, which once combined with appropriate PCB-based microfluidic networks will provide complete LoPCB platforms.

## Introduction

The concept of a PoC laboratory is not new, however it remains an interesting and challenging aspect of clinical diagnosis^1^. The benefits of PoC laboratories are multidimensional, and in addition to direct PoC applications provide convenient means to analyse biomarkers in populations distant from hospitals or clinical laboratories. Moreover, with the sustained expansion of the Internet of Things (IoT), PoC platforms can easily connect to core laboratories and request any required data or processing support. Over the last decade, the rapid diagnosis of TB in both developed and developing countries has benefited from the cost-effectiveness of various POC platforms. However, TB remains one of the world’s deadliest diseases, with one third of the world’s population affected. Globally, in 2014, 9.6 million people became infected, resulting in 1.5 million TB-related deaths^2^.

For PoC devices, the obvious route is to port standard immunochemical assays from the laboratory or “assay track” into PoC diagnostic tools^3–10^. However, certain barriers have prevented wide scale application of PoC technologies. Current assay protocols often require colorimetric detection and thus bulky spectroscopic equipment, and bespoke manufacturing has been required for complex microfluidics and pumps^11, 12^. Versatility has also been an issue; numerous different systems for numerous different assays would clearly not be appropriate for PoC diagnostics as the physician must have a high level of confidence in their tools, gained through repeated application.

There are many reasons why PoC diagnostics have yet to become mainstream among developed economies. Not least are issues in the transfer of complex technologies such as pumps, fluidics and sophisticated detection cells and electrodes to commercial manufacturing processes^13^. To address this issue, our philosophy is to develop highly sensitive assay technology using exclusively commercial PCB manufacturing processes. Our industrial partner, Newbury Electronics, manufacture the prototype PCBs to include fully embedded microfluidic architecture and Au/Ag amperometric electrodes at the PCB surface. This commercially manufactured PCB approach allows the production of completely functional amperometric PCB-based sensing units for a few pence each (excluding the electronics unit and the chemical reagents). Moreover, our design and technology allows any assay adapted to this system to be performed on the standard PCB unit simply by switching reagents^14–16^.

In this publication it is our intention to demonstrate the transition of electrochemical analysis techniques to commercially manufactured surfaces. We present a detailed investigation of the integration points necessary to develop working biodetection systems and progress to a fully working device in partnership with existing manufacturing technologies. We show entirely novel PCB designs, improving sensitivity over commercial colorimetric and published amperometric systems, combined with detailed analyses of pre-calibration performance with reference specifically to clinical diagnostics.

The assay we have implemented is a commercially available anti-Interferon gamma (IFN*γ*) ELISA from R + D Biosystems. IFN*γ* is a pro-inflammatory cytokine with a central role in innate and acquired immunity. In the clinical laboratory interferon gamma release assays (IGRA’s) are used routinely in at risk populations to diagnose latent TB infection (LTBI)^17^. These commercial assays are based on the principle that T-cells in TB patients respond to re-stimulation with TB specific antigens (CFP-10, ESAT-6 and TB7.7) by producing IFN*γ*. IFN*γ* producing T- cells are quantified by ELISAspot (T-Spot TB Oxford Immunotec) or IFN*γ* secretion detected by ELISA (QuantiFERON-TB Gold In-Tube, Cellestis)^18, 19^. However, IGRA’s are time consuming, requiring 12^+^ hours pre-incubation of blood with TB antigens before blood separation. Furthermore, QuantiFERON test samples are usually batch tested for cost-effectiveness, increasing handling times to up to or over one week.

Previously published research work from our group detailed early results using PCB-based assay technology for the detection of IFN*γ* using a commercial potetiostat and provides comparative results from other colorimetric and amperometric endeavours^20^. We now demonstrate improved assay sensitivity over both previously published systems and standard colorimetric methods. In addition, we introduce and apply our own, bespoke, custom-made data acquisition electronics for the PCB-sensors’ read-out. We characterise the sensor performance and identify automated PoC device relevant measurement protocols, in addition to providing individual assay, and individual sensor performance data. Assays are independently characterised by Surface Plasmon Resonance (SPR), and all electrochemical measurements are conducted using bespoke in-house portable electronics. We also demonstrate assay functionality in a blood plasma matrix to reflect the final intended application.

Our commercial PCB manufacturing technologies will lead to the development of a lightweight, low-cost amperometric detection unit, manufactured using standard commercial processes to include embedded microfluidics and multi-channel amperometric sensing. The assays are based on electrochemical detection of 3,3′,5,5′-Tetramethylbenzidine (TMB), a common colorimetric reporter reagent in standard ELISAs. We demonstrate assay component ligand binding by standard SPR to allow comparison to other common assay systems and to evaluate binding of capture antibodies (Cysteine modified ScFv) via thiol link to a Au surface. We show TMB substrate conversion dependent amperometric signal elevation with a linear correlation, and we demonstrate full assay performance in both aqueous and plasma matrices. This PCB technology is simple and many industry standard diagnostic assays could be adapted to this assay platform.

## Results

### Assay Component Analysis

**Figure 1:**
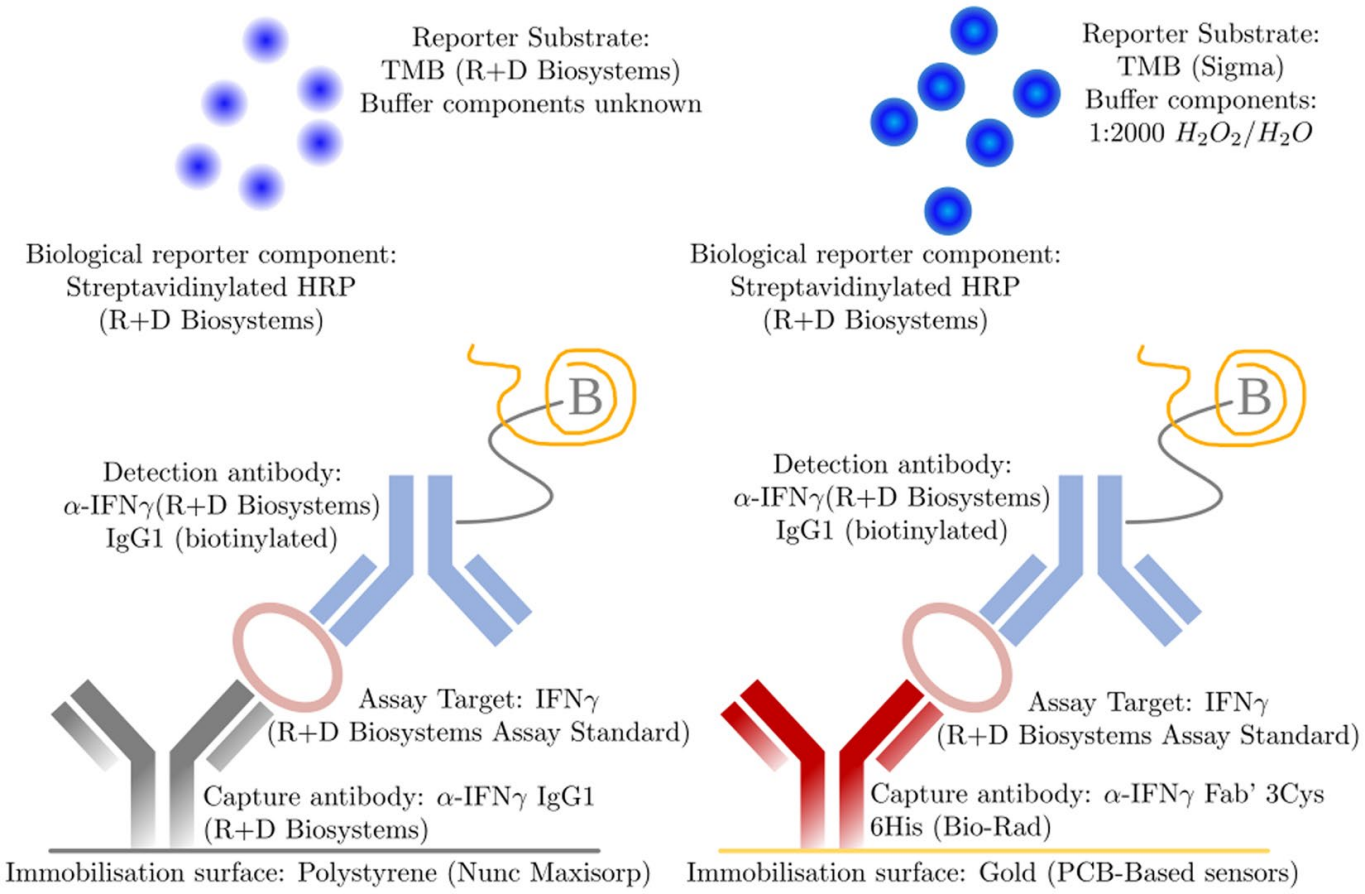
Biological components of the original colorimetric diagnostic assay system (R + D Biosystems) and the converted assay been used for the eELISA detection method.

With the intention of producing a complete hand-held biosensor for the detection of IFN*γ*, the detection range was focused on the clinically relevant range associated with *Mycobacterium tuberculosis* infection. An existing colorimetric diagnostic assay system was adapted to operate with reduced volume, with assay components localised on Au sensor surfaces. The original colorimetric assay system is produced by R + D Biosystems and is capable of identifying IFN*γ* in blood plasma samples with a published detection limit of 15 pg/mL. This commercial system uses standard ELISA analysis of 100 *μ*L volumes in standard 96-well plates. The modified assay operates in 10 *μ*L volume on a Au surface with one major component substitution and further reagent adjustments. Assay changes are illustrated in Fig. 1.

**Figure 2:**
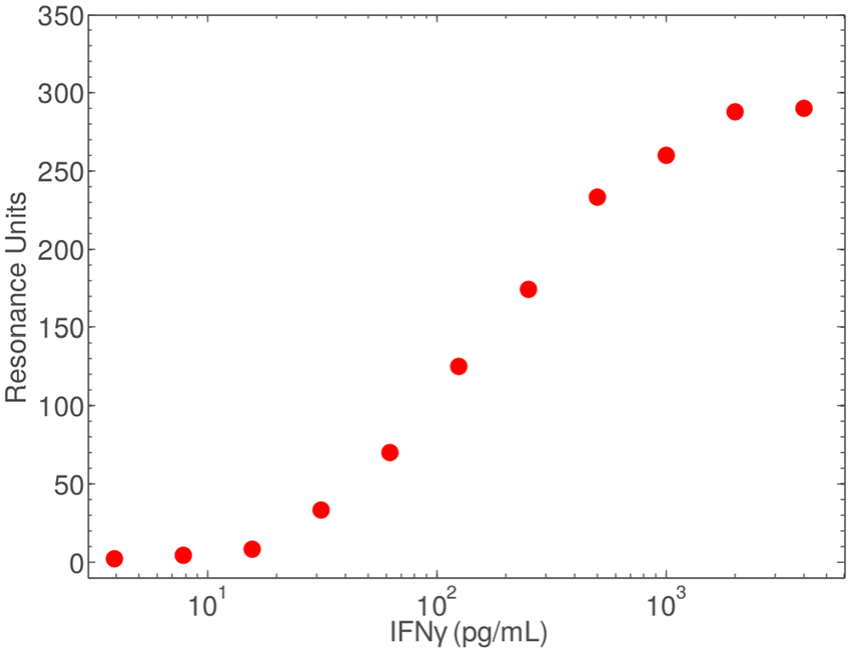
Characterisation of antibody-target interaction using SPR. A standard sigmoid binding curve is produced indicating standard 1:1 binding kinetics.

Initially we characterised antibody-target interaction by SPR. Capture antibody *α* IFN*γ* Fab′-3Cys-6His) was immobilised on a Au sensor chip surface. Covalent binding was achieved via thiol linkage to sulfhydryl groups present due to the modified inclusion of Cysteine residues. This binding step was characterised by SPR, although it must be noted that surface linkage in SPR experiments is conducted under flow, while in the case of our prototype sensor surfaces the linkage step is static. We flowed a series of titrated IFN*γ* samples over the surface of the chip and recorded changes in resonance units (RU) before and after sample flow. The binding characterisation plot is shown in Fig. 2. A standard sigmoid binding curve is produced indicating standard 1:1 binding kinetics. Primarily these results demonstrate that we are able to bind our Cysteine modified Fab′ antibodies passively to a Au surface and then conduct a quantitative assay.

### Sensor Characterisation

The major innovation in our PCB-based hand-held assay is the use of standard low-cost commercial manufacturing techniques. As with electrical connections and microfluidics our electrode surfaces are laid down as part of this process. A thin layer of Cu foil is laminated to the FR4 PCB substrate with heat and adhesive. Then appropriate electrical connections (tracks) and electrode pads are patterned by etching the Cu layer. Next a Au layer is plated onto the Cu surface, fixed by the copper primer which determines the final distribution of Au. In general, electrochemical detection uses highly refined and precisely manufactured electrodes with surface area, volume and geometry designed to optimise electrochemical detection within the specific framework provided by the measurement system. The detection process can then be optimised through electrode design. In order to achieve good precision in detection with titrated samples separated neatly within appropriate standard deviations, a relatively high electrode bias was used (−300 mV), as determined by cyclic voltammetry (CV). This electrode bias provides sufficient resolution between titrated samples, without damaging the electrode surface, since very high biasing voltages may lead to accelerated corrosion of the electrodes due to redox reactions.

**Figure 3:**
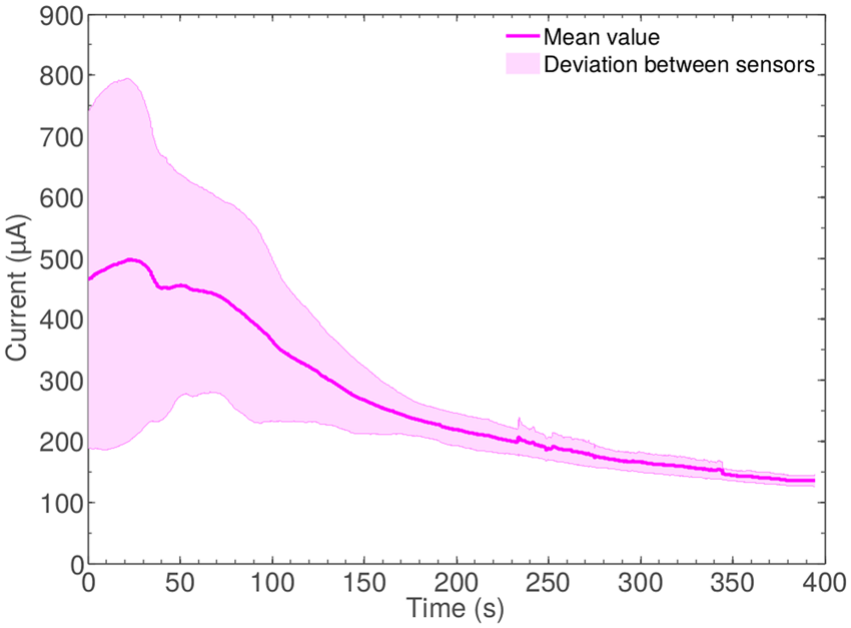
Transient sensor behaviour using a TMB electrolyte (OD 0.5 by colorimetric analysis). All sensors converge to an equilibrium point after a threshold time value (>400 s), which has been selected as a starting point for each measurement herein.

To optimise consistency in signal magnitude between sensors, we characterised sensor activity across extended measurement times to establish appropriate measurement points and periods. 1 M Sodium Chloride (NaCl) was used as the analyte electrolyte and measurement was performed using five different sensors over an extended time period (400 s). This analysis was conducted without voltammetric surface preparation by CV. As it can be seen in Fig. 3, our results showed considerable variability, which would be lead to large errors in quantitative amperometric measurement. However, after 200 s sensor readings converged and signal drift gradually decreased until a tight signal grouping was observed at around 400 s. All measurements presented herein were acquired following a similar electrode conditioning period. The final hand-held PoC sensor design will have access to fluidic control with internal reference standards to calibrate signal levels between sensors.

### Electrochemical TMB Quantification

The amperometric detection methods used in this investigation are often referred to as second generation amperometry^21^. The system directly detects charge carrier concentration through measurement of total current magnitude rather than utilising electrochemical reactions directly at the electrode. The assay components required for integration therefore include only a charge carriage reporter molecule within a relatively low conductivity electrolyte. A very common colorimetric reporter system in standard diagnostic ELISAs is TMB. Reporting functionality is achieved through enzymatic conversion to the corresponding diimine product by the horseradish peroxidase enzyme (HRP). TMB is a colourless liquid, while the resulting product is bright blue. Hydrogen peroxide (H_2_O_2_) is required as an enzymatic co-factor and is consumed in the conversion process providing free hydroxyl ions to the solution^22^. Therefore, this system delivers two charge carriers, a TMB diimine product and free hydroxyls, both of which are theoretically electrochemically active. Our assay system is optimised to detect the TMB product due to the more reactive and transient nature of hydroxyl ions, providing a higher consistency in assay-dependent reporter concentration over time.

**Figure 4:**
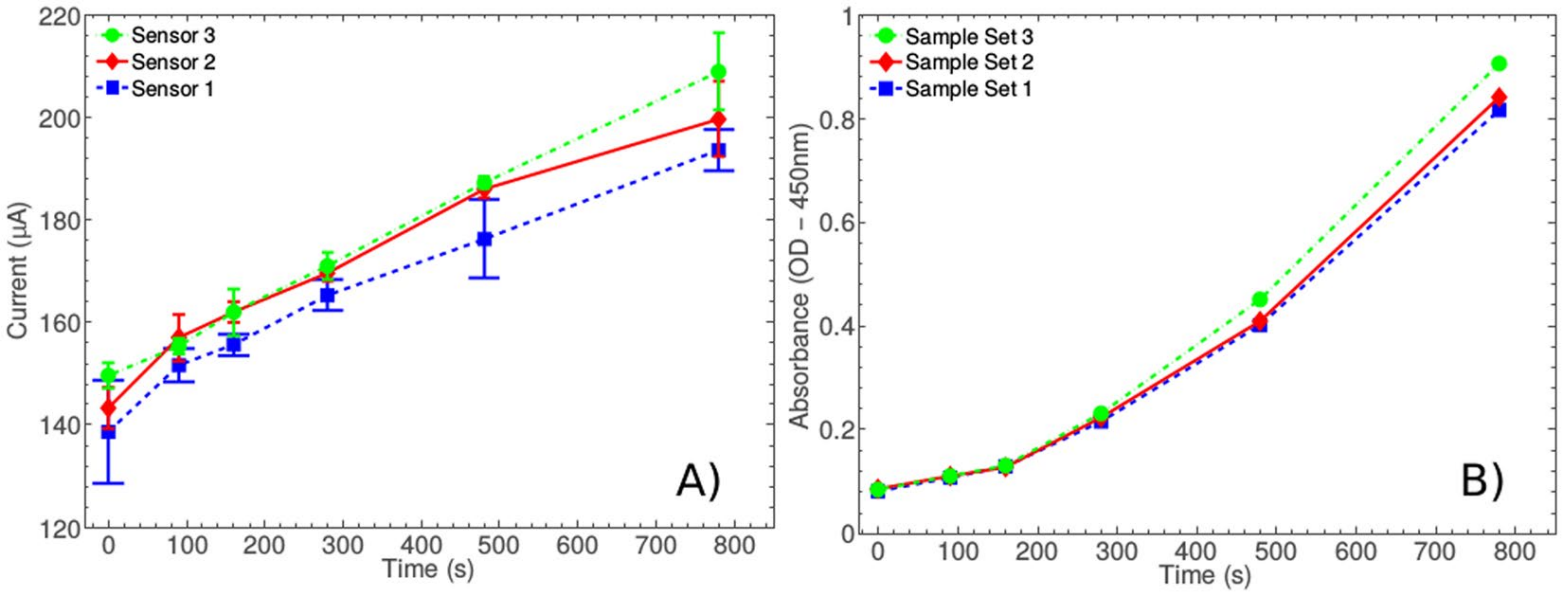
Comparison of amperometric and colorimetric detection of TMB product concentrations. A range of TMB product concentrations were achieved by allowing a range of enzymatic conversion times using a single HRP enzyme concentration. Results were obtained using three different amperometric sensors, however, all colorimetric results were obtained using the same colorimetric quantification apparatus (Promega Glomax).

To establish detection ranges and to prove the concept of TMB we first compared amperometric measurements with colorimetric, using a range of TMB product concentrations. Due to the possibility of the enzymatic component skewing returned amperometric signal we made efforts to minimise electrochemical contributions from this source. Solutions of variable TMB product concentrations were made by adding a measured quantity of HRP to a stock sample of TMB substrate. 200 *μ*L aliquots were then removed at various time-points following reaction initiation by addition of HRP. The reaction was stopped by addition of 20 *μ*L of 1 M HCl, attenuating enzyme activity. A lower pH also reduces the ionic interaction between associated pairs of the diimine product changing the colorimetric absorption maxima from 650 nm (blue) to 450 nm (yellow). The TMB product was then assessed both amperometrically and colorimetrically. Nine samples were measured using three sensors. The results shown in Fig. 4 demonstrate a clear correlation between TMB product concentration as determined by time of reaction, and amperometric and colorimetric signals. Both within sensor variability and between sensors variability fall within acceptable ranges.

Amperometric and colorimetric signals are broadly comparable (see Fig. 4), although the amperometric system does not show signal extinction at lower concentration ranges as seen in the colorimetric analyses. Signal extinction occurs where there are insufficient concentrations of reporter component to register a signal. This effect is more thoroughly characterised in the following sections, where we performed full assay chemistry to measure TMB concentrations from very low target concentrations. These results show that amperometric detection would be as effective as colorimetric for many diagnostic applications. They also demonstrate the suitability of our commercially manufactured PCB-based technology to this task.

### Assay at PCB Surface

The PCB-based detection system uses Au surface to capture and immobilise antibody fragments via Cysteine (thiol) linkage. Three Cysteine residues are conjugated to Fab′ antibody fragments to yield sulfhydryl groups for thiol linkage to the Au surface. The assays were performed at the PCB surface, with proteins immobilised on Au-coated Cu pads. Fluid wells were cut from PMMA and fixed to the PCB surface. Samples were measured electrochemically by removal to a sensor well as described for TMB detection. Colorimetric values were obtained by measuring absorbance at 450 nm using a nanodrop spectrophotometer.

**Figure 5:**
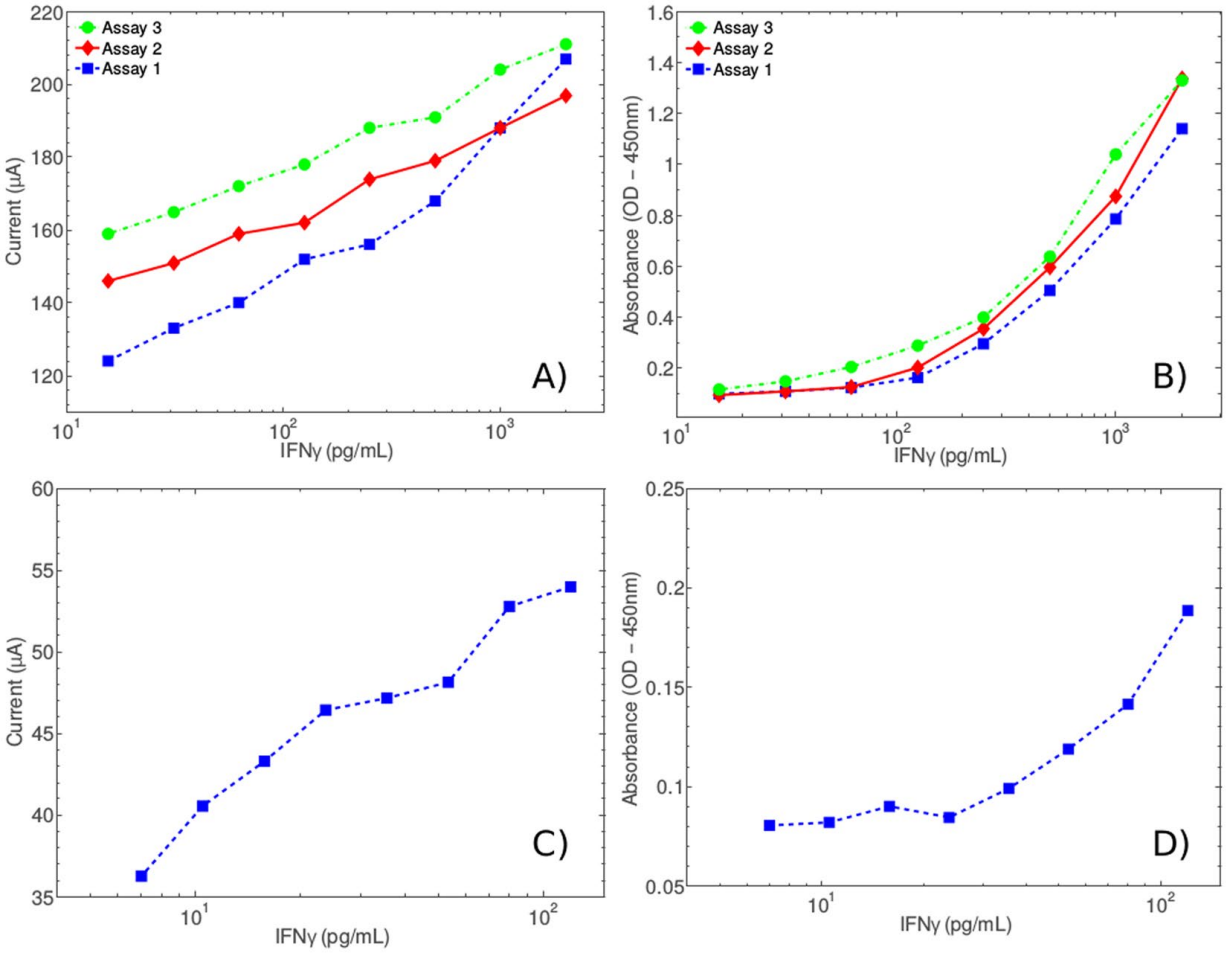
Amperometric assay experiment using the modified assay components measured with the PCB-based sensor. (**A**) Amperometric measurement using the PCB-based sensor and in-house bioinstrumentation platform. (**B**) Colorimetric validation of the experimental results shown in (**A**). (**A**,**B**) Show three discrete IFN*γ* titration assays as described in Fig. 1 with amperometric values measured on three discrete sensors and colorimetric values measured on one sensor (Promega Glomax). (**C**) Amperometric data for low concentration IFN*γ* assay using Log_1.5_ analyte dilutions. (**D**) Colorimetric verification of the experimental results shown in (**C**). Although the colorimetric system demonstrates a LoD of 35 pg/mL for the specific experiment, the amperometric LoD is significantly lower at ~10 pg/mL. Judging from the obtained current ranges and given the characteristics of the bioinstrumentation board, these measurements predict that the LoD could potentially reduce even more in the final optimised system.

Three identical assays were performed and measured using three different sensor surfaces. The results in Fig. 5 show that IFN*γ* detection target levels correlate linearly with the amperometric signal. This assay was able to detect IFN*γ* at a range of concentrations broadly reflecting the clinically relevant range measured in IGRA’s for latent TB infection (10–2000 pg/mL). Linear correlation extends to lower concentrations for the amperometric detection system, where signal extinction is observed for colorimetric analysis.

### High Sensitivity Assay

To investigate our observation that amperometric detection of TMB product is more sensitive than the standard colorimetric system we performed a second assay using lower concentrations of IFN*γ* (10–120 pg/mL), prepared using a shallower dilution scheme. Assay sensitivity for the colorimetric system was determined to be 35 pg/mL, while no sensitivity limit has been effectively demonstrated here for amperometric detection. It should be noted that the lowest concentration sample displayed in Fig. 5 is a blank (0 pg/mL IFN*γ*) and shows the baseline signal, suggesting that the concentrations applied in this experiment are close to the sensitivity level for amperometric detection as part of this assay system. We note that the colorimetric assay is un-optimised, bespoke buffers are supplied with the R + D Biosystems kit upon which this assay is based, these have not been used for this assay. However, our conclusion is that our amperometric system can effectively detect lower levels of TMB product than the colorimetric systems employed herein irrespective of specific assay chemistry. We show that our amperometric system can detect TMB product levels that cannot be detected colorimetrically thus, suggesting that sensitivity can be increased globally for standard colorimetric diagnostic procedures by replacement with amperometric detection. Currently clinical diagnosis of TB by IFNGR assay can only provide diagnosis by recognition of an elevated level of IFN*γ* as normal non-clinical serum levels of IFN*γ* are not detectable. The improved sensitivity we demonstrate in lower concentration ranges could provide significant improvements to diagnostic certainty.

### Assay in Plasma Matrix

**Figure 6:**
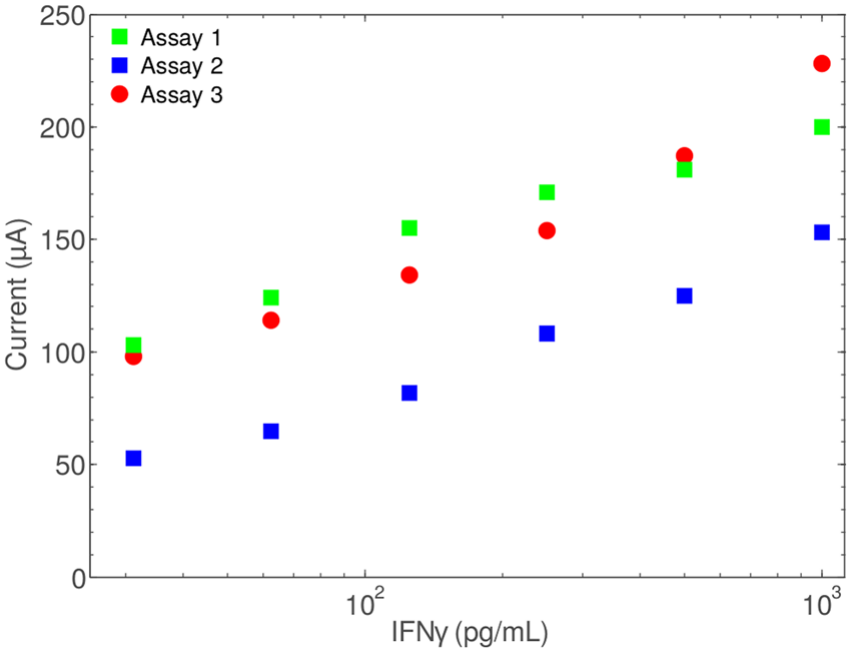
Three discrete IFN*γ* titration assays were performed in a plasma matrix. TMB reporter solutions harvested from each were applied to a different sensor to produce the three measurement series. Therefore, this analysis demonstrates the additive variabilities from assay steps, electrochemistry, and between sensor measurements.

In order to demonstrate that our assay system is suitable for the intended medical application we demonstrate that it is functional in a plasma matrix. As IFN*γ* detection in TB diagnostics must be performed at very high sensitivities (10–1000 pg/mL approx.) measurement samples are likely to be undiluted. Therefore, we demonstrate our assay procedure using IFNγ-spiked human plasma. The obtained results of Fig. 6 are broadly similar to those obtained in the parallel assay run in PBS. We conclude that plasma is an acceptable matrix for our assay and detection system.

## Discussion

**Figure 7:**
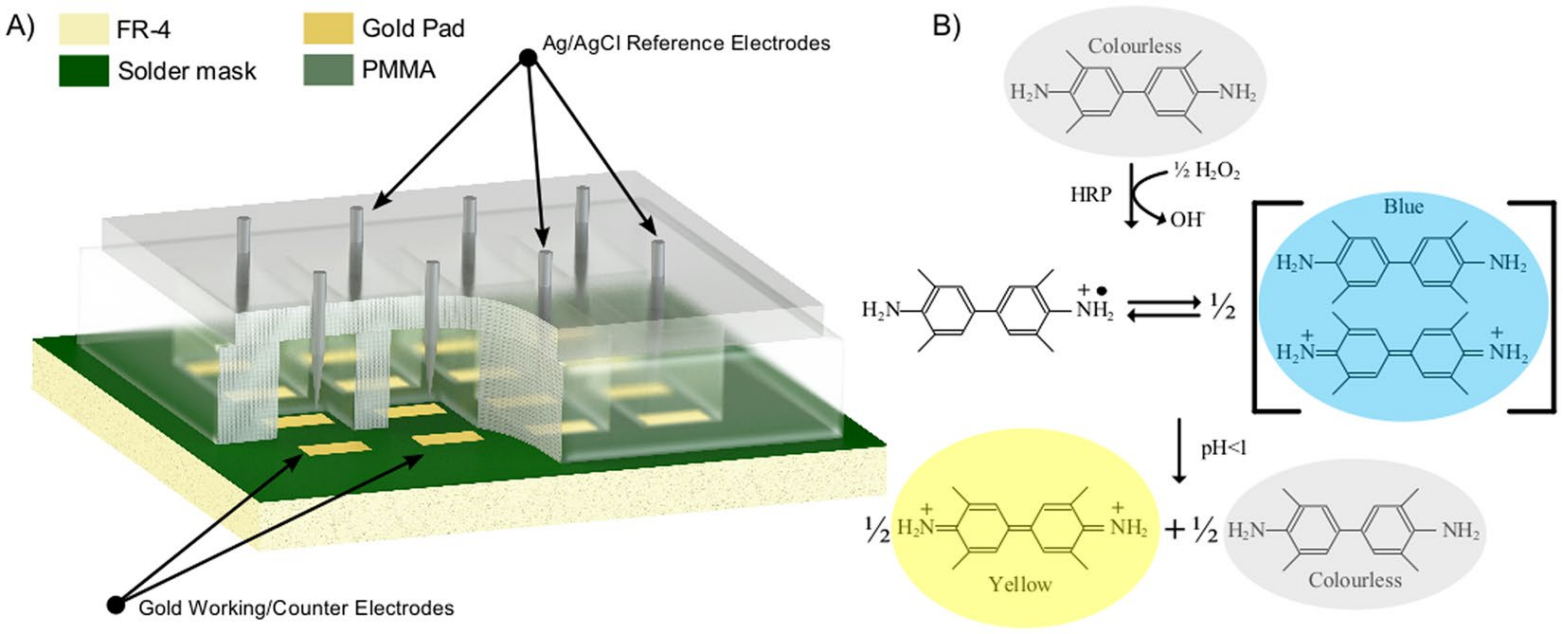
(**A**) A three-dimensional representation of the fabricated prototype PCB-based biosensor and (**B**) the HRP-catalysed oxidation of TMB.

**Figure 8:**
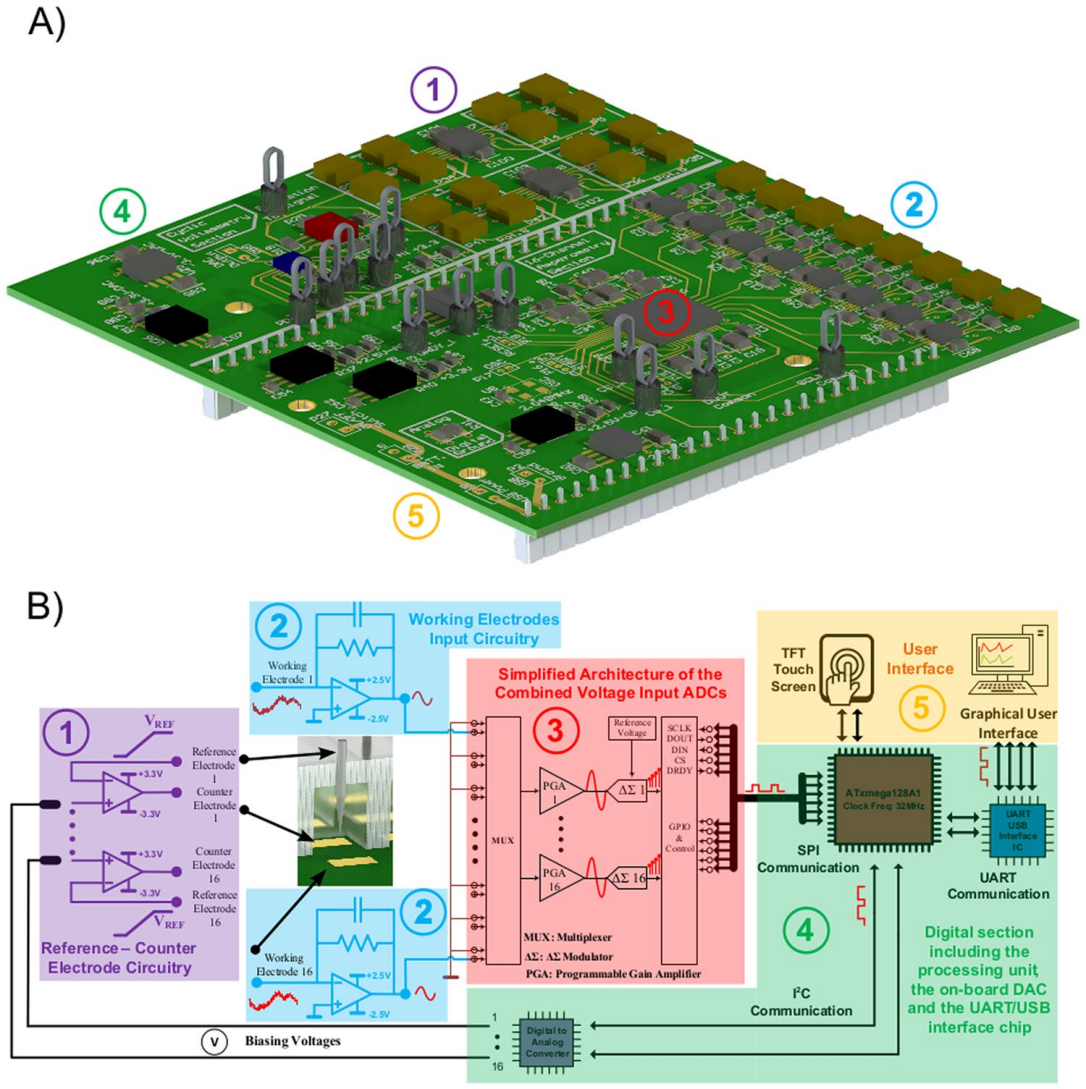
(**A**) A three-dimensional representation of the custom-made electronic board for sensitive amperometric measurements. (**B**) The individual compartments of the electronic board and their approximate location on (**A**).

This work demonstrates components for a framework system for PoC diagnostic platform using low-cost commercially manufactured PCB components (summarised in Figs 7 and 8). As an imminent future step, we seek to dramatically reduce the cost of complex microfluidics by integrating microfluidic channel fabrication into standard commercial PCB manufacturing processes. As such this work should be viewed with reference to other published aspects of this endeavour of our research group, such as our recently developed PCB-based passive microfluidic induction and flow systems, and pressure-driven microfluidic serial dilution networks^12^. The work we present here demonstrates functionality in the molecular and electronic aspects of our system (eELISA), including assay technology and sensitivity, sensor characterisation and reporter signal transduction.

The assay we modified to function within our amperometric system is a high sensitivity sandwich ELISA for IFN*γ* detection produced commercially by R + D Biosystems (see Fig. 1). We have demonstrated assay functionality using a capture antibody specifically modified by papain cleavage to yield Fab′ fragments, and tri-cysteinylation to provide sulfhydryl groups for thiol linkage to an Au surface. The suitability of this immobilisation technique to Au is shown through SPR analysis (see Fig. 2). In order to assess our system performance we isolated the TMB reporter system and assessed amperometric and colorimetric measurements ex-assay. Three discrete TMB product concentration series were measured using our in-house electronics, on each of three different sensors. Standard deviations show a significantly greater range of error than colorimetric analysis when the same sample was measured, demonstrating a greater variability in measurement accuracy with the amperometric system, although error is still confined within acceptable limits (see Fig. 4). For example, in Fig. 4, assay point 5 of sensor 1 shows the highest standard deviation within the normal assay range, although this standard deviation range does not overlap with the standard deviation ranges of the immediately higher and lower TMB product concentrations. Between-sensor error ranges will be eliminated by standard sensor calibration protocols such as using a reference sample and measuring the difference between this and the analytical sample. As previously, we note that the colorimetric comparison presented here is not a true comparison as only a single standardised colorimetric sensing system is available.

With respect to the IFN*γ* assay, the results in triplicate of Fig. 5 demonstrate linear signal progression with increasing concentrations of TMB product as determined by enzymatic conversion time. Unique assay points of equal IFN*γ* concentration show some distribution across a range of μA values demonstrating a requirement for individual sensor calibration within the final device but confirming IFN*γ* concentration-dependant signal magnitude suitable for IFN*γ* quantification with these assay components. We note that no comparative assessment of standard colorimetric measurement is available as the colorimetry shown is produced using a single sensing device. Due to the non-uniform nature of our sensing surfaces when compared to standard commercial (and much more expensive) electrochemical electrodes we assessed sensor performance over an extended time period to establish appropriate measurement points and periods. Our data show that while signals are initially very variable, at 400s they converge to a tight grouping at around 140–160 *μ*A. Results presented herein were thus recorded at 400 s across a period of 30 s.

The previous observations made during TMB product detection analyses led to the suggestion that our amperometric detection system may allow identification of even lower concentrations of TMB product than equivalent colorimetric detection (data not shown). For this reason we conducted a very-low-concentration IFN*γ* assay using a shallower IFN*γ* target dilution scheme. We observe ostensible colorimetric signal extinction at 24 pg/mL of IFN*γ*, while amperometric limit of detection (LoD) appears to be at IFN*γ* concentrations of ~10 pg/mL. Finally, the experiment in Fig. 6 performed in a plasma matrix verifies the hypothesis that plasma is an acceptable matrix for our proposed assay and detection system.

Given the very widespread application of standard colorimetric analyses in clinical diagnostic laboratories and hospitals we feel the provision of a reporter detection system able to significantly increase standard assay sensitivities would constitute a medically and commercially significant output. It is noteworthy that the detection system alone can be applied to standard ELISA analysis without altering assay technology that has already been extensively validated through both FDA product confirmation and years of clinical diagnostic application. This is in addition to the provision of a comparable hand-held assay system. The next stage of this ongoing research and development project will be to integrate passive fluidics to include serial dilution and fluid induction^12^ and complete development of fluidic channels to allow switching between analyte and reference samples and achieve reliable signal calibration. We will then demonstrate full diagnostic capability and statistical performance verification through the measurement of a statistically significant cohort of clinical samples.

## Conclusions

We demonstrate an exploded assay system to be considered in concert with passive microfluidic induction and sample handling systems we have developed and published elsewhere^12^. Our systems are fabricated using standard commercial PCB manufacturing techniques which deliver low-volume microfluidic channels, and integrated sensor and assay surfaces. We combine the advantages of low-cost lateral flow diagnostics with high-value high-sensitivity microfluidic PoC systems. We have demonstrated applicable assay chemistry by converting an industry standard high-sensitivity IFN*γ* assay to our low volume integrated assay and sensor pads. We have further demonstrated improved detection sensitivity of our amperometric system when compared to the standard colorimetric equivalent theoretically allowing sensitivity improvements in all TMB-reporter colorimetric assay systems. We have also characterised sensor performance and measurement point and period. Next we intend to assemble a working prototype with full microfluidics and demonstrate a fully automated hand-held PoC assay system.

## Materials and Methods

### Surface Plasmon Resonance

Anti-IFN*γ* Fab′(Cys)3 antibodies were diluted to 20 *μ*g/ml in PBS. The sample was flowed across a one channel of standard Au SPR chip (25 *μ*L/min, 5 minutes), while the other channel was concurrently treated with 100 mM HEPES system buffer to act as a measurement blank. A Log_2_ IFN*γ* titration was prepared in PBS with a top concentration of 4 ng/mL. Samples were flowed across the prepared chip surface channels (25 *μ*L/min, 4 minutes) and RU spectra recorded. Chip surfaces were regenerated between sample measurements via pH desorption using 100 mM Phosphoric acid (25 *μ*L/min, 4 minutes). Displayed values (see Fig. 2) were prepared by subtracting blank values from the reference channel from the measurement channel and recording the difference in RU between before sample flow induction and after sample flow cessation and observed equilibration.

### TMB Detection

Samples of varying TMB product concentration were prepared by adding 1 *μ*L 1 mg/mL HRP (R + D Biosystems) to 10 mL of TMB substrate (1 TMB tablet (Sigma Aldritch T5525) in 10 mL de-ionised water and 5 *μ*L 30% (by volume) H_2_O_2_). 1 mL aliquots were then removed at recorded time-points and the enzymatic reaction stopped immediately in the removed aliquot by addition of 100 *μ*L 1 M HCl. 40 *μ*L samples were measured in bespoke PCB-based cells consisting of an internally fabricated PMMA well (50 *μ*L total volume) over two Au surface electrodes and an introduced Ag|AgCl reference electrode (Ag chlorinated by immersion in 30% NaClO for 15 minutes). Measurements were taken using the described in-house electronic control board.

### Standard Amperometric Assay

Assays were performed in previously described bespoke measurement cells fixed at the PCB surface. Gold electrodes were used to localise cysteinylated Fab′ antibodies via covalent thiol linkage directly to the Au surface. Fab′ antibodies were prepared at a concentration of 40 *μ*g/mL in PBS and 10 *μ*L incubated overnight in each assay cell at 4 °C. Assay wells were rinsed twice with PBS and 50 *μ*L of 1% BSA (W:V) in PBS introduced to each well to block exposed hydrophobic surfaces. Blocking was allowed to progress for 2 hours at RT. Wells were rinsed twice with PBST (1xPBS, 0.05% tween_20_ (by volume)) and 20 *μ*L of titrated IFN*γ* was added. The IFN*γ* titration was prepared in Log_2_ dilution in PBS across 8 assay points from a top concentration of 2 ng/mL. IFN*γ* incubations were allowed to continue for 1 hour at RT. Wells were washed twice with 50 *μ*L PBST and once with 50 *μ*L PBS. Biotinylated detection antibody (R + D Biosystems) was prepared at 200 ng/mL in PBS and 20 *μ*l added to each well. Incubations were allowed to progress for 1 hour at RT. Wells were washed twice with 50 *μ*L PBST and once with 50 *μ*L PBS. Streptavidin::HRP was prepared in PBS at a dilution of 1:20 from the R + D Biosystems kit stock (no data provided for concentration). 20 *μ*L of streptavidin::HRP working dilution was added to each well and incubated for 15 minutes at RT. Wells were washed twice with 50 *μ*L PBST and once with 50 *μ*L PBS. TMB substrate was prepared using stock tablets (Sigma-Aldritch T5525) dissolved in 10 mL of de-ionised water containing 5 *μ*L 30% (by volume) H_2_O_2_. Standard buffers (Phosphate-Citrate) were omitted from the TMB solution to minimise background electrochemical signal and minimise buffering of relevant electrochemically active species. The enzymatic reaction was allowed to proceed for 20 minutes at RT with light excluded. 40 *μ*L samples were measured in bespoke PCB-based cells consisting of an internally fabricated PMMA well (50 *μ*L total volume) over two Au surface electrodes and an introduced Ag|AgCl reference electrode (Ag chlorinated by immersion in 30% NaClO for 15 minutes). Amperometric measurements were taken using the described in-house electronic control board providing a reference electrode bias of −300 mV. Colorimetric analysis was conducted using a Promega GloMax spectrophotometer recording at 450 nm.

### High-Sensitivity Amperometric Assay

High-sensitivity assays were performed in standard Nunc Maxisorp 96-well plates. Fab′ antibodies were localised at the well surface via hydrophobic partitioning. Fab′ antibodies were prepared at a concentration of 2 *μ*g/mL in PBS and 50 *μ*L incubated overnight in each assay well at 4 °C. Assay wells were rinsed twice with PBS and 300 *μ*L of 1% BSA (W:V) in PBS introduced to each well to block exposed hydrophobic surfaces. Blocking was allowed to progress for 2 hours at RT. Wells were rinsed twice with PBST (1xPBS, 0.05% tween_20_ (by volume)) and 60 *μ*L of titrated IFN*γ* was added. The IFN*γ* titration was prepared in Log_1.5_ dilution in PBS across 7 assay points and one blank from a top concentration of 120 pg/mL. IFN*γ* incubations were allowed to continue for 1 hour at RT. Wells were washed twice with PBST and once with PBS. Biotinylated detection antibody (R + D Biosystems) was prepared at 200 ng/mL in PBS and 70 *μ*L added to each well. Incubations were allowed to progress for 1 hour at RT. Wells were washed twice with PBST and once with PBS. Streptavidin::HRP was prepared in PBS at a dilution of 1:20 from the R + D Biosystems kit stock (no data provided for concentration). 80 *μ*L of streptavidin::HRP working dilution was added to each well and incubated for 15 minutes at RT. Wells were washed twice with PBST and once with PBS. TMB substrate was prepared using stock tablets (Sigma-Aldritch T5525) dissolved in 10 mL of de-ionised water containing 5 *μ*L 30% (by volume) H_2_O_2_. Standard buffers (Phosphate-Citrate) were omitted from the TMB solution to minimise background electrochemical signal and buffering of relevant electrochemically active species. The enzymatic reaction was allowed to proceed for 20 minutes at RT with light excluded. 30 *μ*L samples were measured in bespoke PCB-based cells consisting of an internally fabricated PMMA well (50 *μ*L total volume) over two Au surface electrodes and an introduced Ag|AgCl reference electrode (Ag chlorinated by immersion in 30% NaClO for 15 minutes). Amperometric measurements were taken using the described in-house electronic control board providing a reference electrode bias of −300 mV. Colorimetric measurements were taken using a Nanodrop spectrophotometer.

### Assay in Plasma Matrix

The assays conducted under plasma sample matrix were performed using the same protocol applied to standard assays in PBS with the exception that IFN*γ* standards were diluted into plasma.

### Sensor Characterisation

40 *μ*L of 1 M NaCl was used as a reference sample in each of five previously described bespoke electrochemical measurement cells. The described in-house control board supplying a reference electrode bias of −300 mV was used to collect amperometric data over an extended measurement period of 400 s.

### The 16-channel Bio-Instrumentation Board

A custom-made, 4 layer Printed Circuit Board (PCB) has been designed and fabricated for the appropriate biasing of the PCB-based amperometric sensor and for the concurrent amperometric signal detection (see Fig. 8). The bioinstrumentation board is providing 16 current input channels (where working electrodes are connected) and 32 reference and counter electrode connections (16 connectors each). The captured input current is converted into a voltage signal using standard transimpedance amplifier topologies (LTC2055 operational amplifier from Linear Technologies) with high accuracy feedback resistors (0.1% tolerance) and is filtered from high-frequency noise components using appropriate value capacitors. Subsequently, two, fully-differential eight channel, 16-bit resolution voltage-input Analogue-to-Digital Converters (ADCs) ADS1198 (Texas-instruments) are employed for the digitisation of the incoming voltage signals^23^. The ADCs are operating with bipolar power supply (±2.5 V) and therefore, are able to detect both reduction and oxidation reaction currents. The overall design of the board revolves around the idea of immediate digitisation of the analog biosensor signals, process them by means of standard Digital Signal Processing (DSP) techniques and finally send them to the user in digital form either to a PC or to the embedded on-board TFT touch screen^24, 25^. The appropriate biasing voltages for the reference electrodes are provided by a two-wire interface, 12-bit, single channel Digital-to-Analog Converter (DAC), the AD5321 (Analog Devices) coupled with an OP295 (Analog Devices) operational amplifier-based circuit topology, which allowed bipolar operation of the DAC. Finally, 4 high-precision, bipolar power supply, low offset voltage quad amplifiers LMP7704 (Texas Instruments) have been placed as potentiostats for the three-electrode amperometric measurement. The total current consumption of the electrical platform does not exceed 80 mA, which translates into an ~30 h full operation from a low-cost commercially available 3.7 V, 2500 mAh Li-Ion rechargeable battery.
